# Factors perceived to influence rural career choice of urban background family physicians: A qualitative analysis

**DOI:** 10.36834/cmej.56976

**Published:** 2020-07-15

**Authors:** Olga Szafran, Douglas Myhre, Jacqueline Torti, Shirley Schipper

**Affiliations:** 1 Department of Family Medicine, University of Alberta, Alberta, Canada; 2 Department of Family Medicine, Cumming School of Medicine *and at the time of the study was* Associate Dean, Distributed Learning and Rural Initiatives, University of Calgary, Alberta, Canada; 3 *At the time of the study* Department of Family Medicine, University of Alberta. Alberta, Canada. *Presently* Centre for Education Research and Innovation, Schulich School of Medicine and Dentistry, Western University, Ontario, Canada; 4 Faculty of Medicine & Dentistry *and* Associate Professor, Department of Family Medicine, University of Alberta, Alberta, Canada

## Abstract

**Background:**

Urban background physicians are the main source of physician supply for rural communities across Canada. The purpose of this study was to describe factors that are perceived to influence rural career choice and practice location of urban background family medicine graduates.

**Methods:**

We conducted a qualitative, descriptive study employing telephone interviews with 9 urban background family physicians practicing in rural locations. Those who completed residency training between 2006 and 2011, were in rural practice, and had an urban upbringing were asked: when the decision for rural practice was made; factors that influenced rural career choice; and factors that influenced choice of a particular rural location. Emerging themes were identified through content analysis of interview data.

**Results:**

We identified four themes as factors perceived to influence rural career choice - variety/broad scope of rural practice, rural lifestyle, personal relationships, and positive rural experience/physician role models. We also identified factors in four areas perceived to influence the choice of a particular rural practice location - having lived in the rural community, spousal influence, personal lifestyle, and comfort with practice expectations.

**Conclusion:**

Decisions for rural career choice and rural practice location by practicing urban background family medicine graduates are based on clinical practice considerations, training experience, as well as personal and lifestyle factors.

## Abstract (fr)

**Contexte:**

Les médecins provenant d’un milieu urbain représentent la principale source de médecins pour les communautés rurales au Canada. Cette étude vise à décrire les facteurs qui sont perçus comme influençant un choix de carrière en milieu rural pour les diplômés en médecine familiale provenant d’un milieu urbain.

**Méthodes:**

Nous avons mené une analyse qualitative et descriptive à l’aide d’entrevues téléphoniques avec neuf diplômés en médecine familiale provenant d’un milieu urbain. On posait les questions suivantes à ceux qui avaient terminé leur résidence entre 2006 et 2011, qui pratiquaient en milieu rural et qui avaient grandi en milieu urbain : à quel moment la décision de s’établir en milieu rural a-t-elle été prise? Quels sont les facteurs qui ont influencé le choix de carrière en milieu rural? Quels facteurs ont influencé le choix d’un emplacement rural particulier. Les thèmes émergents ont été relevés par l’analyse du contenu des données d’entrevue.

**Résultats:**

Nous avons répertorié quatre thèmes comme facteurs perçus pour influencer le choix d’une carrière en milieu rural : la variété et la portée élargie d’une pratique en milieu rural, le style de vie rural, les relations personnelles et des expériences rurales positives/modèles de rôle de médecin en milieu rural. Nous avons également relevé quatre thèmes perçus pour influencer le choix de s’établir dans un milieu rural particulier : avoir vécu dans cette communauté rurale, l’influence du conjoint, le style de vie personnel et se sentir à l’aise avec les attentes de ce milieu de pratique.

**Conclusion:**

Les décisions d’un choix de carrière en milieu rural par des diplômés en médecine familiale provenant d’un milieu urbain et maintenant en pratique, sont fondées sur des considérations de pratique clinique, l’expérience de la formation ainsi que des facteurs personnels et de style de vie.

## Introduction

The shortage of physicians in rural areas is a continuing challenge in North America.^1^^,^^2^^,^^3^ Strategies to increase rural physician supply have had little apparent impact over many years.^4^ The decision to pursue a rural career is ultimately made by the physician who must consider a multitude of personal and professional factors. The geographic background of a physician has been shown to be a predictor of practice location, with physicians with a rural background,^5^^,^^6^^,^^7^^,^^8^^,^^9^ those who either have a rural background spouse/partner^6,^^10^ or a spouse receptive to a rural lifestyle^7,^^11^ being more likely to choose a rural practice. While rural background is a strong predictor of rural practice,^12^^,^^13^ studies have reported that between one-third to two-thirds of rural physicians in fact come from an urban background,^14^^,^^15^^,^^16^^,^^17^ and as such, are an important source of physician workforce for rural areas. Urban background physicians who choose a rural career path have been labelled as “convertibles.”^18^

A scoping review of determinants of urban background medical students choosing rural practice grouped influential career decision factors into four theme areas: scope of practice and personal satisfaction, rural training, premedical school mindset to practice rurally, and economic factors.^19^ Medical students from an urban background appear to be attracted to the broad scope of rural practice which they believe provides personal satisfaction, a challenging career, and better establishment of the doctor-patient relationship.^14^^,^^19^ Undergraduate and postgraduate rural training experiences are influential factors in rural career choice by expanding the horizons of urban background learners and exposing them to rural culture and rural patients.^20^^,^^21^ The duration of rural training has been positively associated with rural practice location,^14^^,^^23^ thus, early and substantial exposure to rural practice in medical training is important for expanding the outlook of urban background learners, experiencing the diverse scope of rural practice, and gaining an increased understanding of rural culture.^19^^,^^22^ Some urban background students appear to have a predetermined career preference for rural practice,^24^ particularly those who have a partner from a rural background, and are more likely to have a mindset to choose rural practice.^10^ Debt repayment or financial incentives have been reported to motivate some urban background physicians to practice rurally, particularly those with substantial student debt or other financial stressors.^14^ Whether these factors also influence urban background family medicine graduates remains to be determined.

Urban background family physicians are a significant source of physician supply for rural communities, yet there is a paucity of research exploring this topic. An examination of the factors that influence rural career choice of urban background family physicians has the potential to inform strategies for recruitment of urban background physicians to rural areas, as well as guide curricular changes for family medicine residency training. As such, the purpose of this study was to describe factors that are perceived to influence overall rural career choice and specific practice location choice of urban background family physicians practicing in rural communities, and to ascertain when these physicians made the decision for rural practice.

## Methods

### Study design & participants

We conducted a qualitative, descriptive study employing interviews with urban background graduates of family medicine residency programs who were in active rural practice. The methodological orientation of the study was that of qualitative, latent content analysis. Study participants completed family medicine residency training at the University of Alberta or University of Calgary between 2006-2011. The qualitative phase was part of a larger study that surveyed graduates about their educational experience during residency training, preparedness for practice, practice location, and scope of practice. A total of 307 out of 651 graduates responded to the survey overall. Those who were of an urban background and had indicated as part of the survey that they would be willing to be contacted to take part in an interview were eligible to participate in the qualitative phase. For the urban background rural practice portion of the study, the sampling frame included 31potential participants.

### Setting

In 2016, within the context of the province of Alberta, rural was considered to include small rural communities, remote communities, and five geographically dispersed regional centres (each having a population of approximately 60,000-100,000). It excluded only the two major cities of Calgary and Edmonton, each with a metropolitan population of approximately one million. As such, we defined urban background as having lived in an area with >200,000 population prior to one’s 18th birthday. Within the scope of this study, we considered practice location to be rural if the population was ≤200,000, for the noted reasons.

### Interviews

One of the authors (JT) conducted telephone interviews during the period November 2015 to March 2016. At the time of the study, JT was a graduate research assistant on the project and had no prior relationship with the participants. Participation was voluntary. We provided all participants a study information letter prior to obtaining written consent before each interview. We used a semi-structured interview guide (Table 1) with open-ended questions to collect data. We audio-taped all interviews and these were subsequently transcribed verbatim by a professional transcriptionist. Interviews ranged from 19 to 45 minutes in duration, with the average being 29 minutes.

**Table 1:** Interview questions

1. When was the decision for rural practice made? a. At what point did you decide to practice in a rural area? *Probes* ***- Before medical school?****If YES, what influenced your decision (shadowing experience, family, recreation, other)? Did you shadow a rural physician or urban physician? Family physician or specialist? What was your shadowing experience like? Describe in detail*. ***- During medical school?*** *If YES, what influenced your decision (rural rotations, longitudinal integrated clinical clerkship, other)?* *What was your first choice of residency program during the CaRMS match (rural family medicine, urban family medicine, specialty)? Were you matched to your first choice? If NOT, what happened?* ***- During residency?****If YES, how was the decision made? What influenced your decision (rural rotations, rural program, family, spouse, other)* ***- After residency?****If YES, how was the decision made? What influenced your decision (locums, job offer, family, spouse, other)?* b. During medical school, did you have rural exposure experience? *If YES, what type of rural exposure (mandatory, elective, integrated clinical clerkship (ICC), family medicine, specialty)? Where (location)? How long (months)? Quality of the rural experience (satisfaction)? Did this experience affect your decision to practice in a rural location?* c. During residency training, did you have rural exposure experience? *If YES, what type of rural exposure (mandatory, elective, remote)? Where (location)? How long (months)? Quality of the rural experience (satisfaction)? Did this experience affect your decision to practice in a rural location?*
2. What factors influenced your decision to practice in a rural area? *Probes - Age of children (if applicable)? Debt repayment options (medical school debt, educational loan)? Family considerations? Family of origin? Financial incentives? Recreational activities for children (dance, piano, skating, etc.)? Recreational activities for self or spouse? Rural lifestyle? Rural physician mentor/role model? Rural rotations during medical school? Rural rotations during residency? Schools for children (if applicable)? Scope of rural practice? Significant other/spousal influence? Other?*
3. What were the key factors affecting your choice of rural practice location? *Probes - How did your experience in the family medicine residency program affect your decision concerning practice location? .Did a particular experience change your mind about practicing in an urban location?. What professional, family and/or other factors affected your decision concerning practice location?*

### Data analysis

Prior to qualitative analysis, we cleaned the transcribed interview data to remove potential participant identifiers and assigned a numeric code to each participant to protect their identity. We manually conducted qualitative content analysis. Each of the authors independently and inductively coded the interview data to identify emerging categories and themes. Subsequently, we held regular team meetings to review each transcript, discuss independent coding, and reach consensus on emerging themes. We created a working document to track independent and team coding and to identify key quotes. This process also acted as a form of audit trail to track study progress and document analytic decisions. This, in addition to analyst triangulation, helped to improve the reliability of the study findings.

## Results

Nine urban background family medicine graduates who were in rural community practice took part in the interviews: six women and three men; seven Canadian graduates and two international medical graduates; five practiced in rural communities of ≤10,000 population, three in communities of approximately 30,000 population, and one in a regional rural community of about 100,000 population. Two urban background graduates completed a rural residency program and the remaining seven completed urban programs.

### Timing of rural career decision

Study participants predominantly indicated that they made the decision to practice in a rural area either during medical school (n=4) or during family medicine residency training (n=3); one made the decision before medical school and one after several years in clinical practice. Those who made the decision during medical school indicated that their undergraduate rural rotation had influenced their decision.

*“…when I was doing my rural rotation … is when I started to get exposed. I think it’s a great thing that most of the universities in Canada [are] getting us to do a compulsory rural rotation, I don’t think I would’ve considered it otherwise … it was an eye opening experience to see my preceptor… doing the full spectrums list, GP, in the clinic, doing rounds at the hospital, doing emerg, doing surgical assist and quite a few things…”* (Interview 6)

For some, personal lifestyle factors and exposure to various specialty rotations during medical school solidified their decision for a rural practice.

*“…I wanted to be able to live in a rural area, my biggest hobby is with horses,… I had realized that the [specialist] lifestyle wasn’t going to work for me long term. Surgery rounds at 6 a.m. and all of that, it was [going to] pay for my horses, but it wasn’t [going to] give me any time for them.”* (Interview 4)

Urban background family physicians in rural practice also indicated that rural exposure during residency training had influenced their decision for rural practice. They noted that the urban residency program allowed for extended rural exposure, such that three urban program graduates had a total of five to seven months of rotations in rural settings (mandatory and elective time combined).

The one participant who had made the decision for rural practice before medical school was influenced by a spouse who did not want a city lifestyle. The other participant who made the decision after several years in urban practice was ready for ”*something new.”*

### Factors influencing rural career choice

We identified four themes as factors that influenced the decision of urban background graduates to practice in a rural area: (1) variety and broad scope of rural practice; (2) rural lifestyle; (3) personal relationships; and (4) positive rural experience and physician role models.

Study participants noted that the *broad scope and variety of rural practice* factored into their decision for choosing rural practice because they saw it as providing: opportunities for broad diversity in clinical practice (e.g. common clinical problems, maternity care, care across the life span, palliative care); opportunities to practice in different settings (e.g. office practice, hospital, emergency department, operating room, long-term care, after-hours clinic ); and more options for professional work (e.g. teaching, clinical work).

*“Well the most important thing was the variety that I could do… emergency,… OR assist clinic, the after-hours clinic. So there was a lot of different things, not only office work. That’s what appealed to me*.” (Interview 5)

*“… inpatient work, emergency medicine, palliative care. It was more the opportunity to actually practice family medicine to its fullest.”* (Interview 8)

Participants also identified that they considered *rural lifestyle* in their practice decision. This included living a simpler lifestyle that lacked the busyness of large cities, being closer to the outdoors and nature, and outdoor recreational activities. In contrast to urban locations, participants frequently commented on the absence of traffic jams and quicker commutes to work as attractive features of rural life.

*“Everything that I need is in town and it’s not a big problem, but the added benefit was less traffic, nice drive from home to work, fresh air, nature. All that is an added bonus.”* (Interview 6)

*“So primarily lifestyle. I don’t like commuting, I like small towns where you really get to know people and it gives access to the outdoors. And so for me, a lot of is the community and the activities outside of medicine that draw me to a small center.”* (Interview 9)

Participants commented that support from *family, and personal and collegial relationships* were an influential factor in deciding to practice in a rural area. Spousal influence was an especially important factor, either because the spouse had a rural background or was attracted to a rural lifestyle and positively introduced their urban background spouse to rural life. In addition, being close to family, as well as having supportive professional rural colleagues, were important considerations in a rural career choice.

*“But certainly being able to support her and just through our years of dating and engagement, I started to fall in love with a whole lot of the aspects of the rural community and the outdoors that she introduced me to, things like backpacking and hiking and just living in a …rural farm setting.”* (Interview 9)

*“So the combination of the nurses that you work with and the other physicians that you work with, it’s a lot tighter of a community and less chaotic of a hospital situation.”* (Interview 9)

Participants indicated that a *positive rural experience and positive rural physician role models* influenced their decision for a rural career choice. This encompassed witnessing a supportive working environment and experiencing positive working relationships among health professionals. Seeing exemplars that role modelled a stimulating and rewarding professional life, as well as a fulfilling personal life, was particularly influential.

*“I think it was more just like their example of their attitude and balance of life and that they were practicing and living in a way that I hoped that I would.”* (Interview 2)

*“Until I got into medicine and understood those doctors really had to work just on clinical skills and suspicion a lot of the time and it’s all I had seen and I had admired them and so I certainly had no problem thinking about working in a rural area.”* (Interview 3)

### Factors influencing location of rural practice

We identified factors that influenced the choice of the specific rural practice location in four theme areas: (1) having lived in that rural community; (2) spousal influence (3) personal lifestyle; and (4) comfort with practice expectations. Financial incentives were not particularly influential in urban background graduates’ decisions to practice rurally.

Study participants noted that the experience of *living in the rural community*, usually during residency training, had given them the opportunity to experience rural practice and rural lifestyle. Within the Alberta context, the rural placements were in smaller communities and not in regional centres. This longer term exposure enabled residents to forge professional and personal relationships and connections within the community, and afforded the opportunity to gain confidence and comfort with clinical rural practice.

*“Well once I had lived here for a few months during my residency rotation, I did love the town, the people, the relationships that I was forming, the nursing staff at the hospital are fantastic, so between our emerg and our acute care, it’s a place that I felt like I would be really comfortable working.”* (Interview 4)

*“Mainly because as I did my residency in [this town], I knew what I could and what I couldn’t… and what would be referred, what needs to be referred and what has to go and so it makes a lot of difference working in that area and then starting your practice in the same area so you know the ins and outs of things there.”* (Interview 5)

Participants commented that *spousal influence* played a significant role in deciding on a specific practice location. Often the rural practice location was determined based on where the spouse grew up, was in close proximity to family, or in proximity to favored recreational activities.

*“…my husband grew up in a small town, so we ended up in this area because this is where he’s from … that’s why we chose to live here…we chose this community in particular, not somewhere else. … I think that he always wanted to come near back his home.”* (Interview 1)

Participants noted that possessing a *comfort level with practice expectations* at a particular clinical practice site was influential in deciding on whether or not to practice at that site. For some, this comfort level included having the clinical confidence to practice rural medicine with limited technological resources. For others, familiarity with practice operations and having the practice organization and management aspects already in place facilitated the decision to practice at that location.

*“I did actually join [this practice] just because there was no need then to take out loans, open a clinic, that type of thing, and his was the one in town that did have space, lots of spaces actually. And there was also basically a ready built patient population to step into, and so the income stream was going to be good,…“* (Interview 4)

*“…no pressure, you know what I really liked about the [clinic] was that they do very easy booking, you know they work from 7 to 5, with 15 minute slots and 2 hour lunch break, so it was a very easygoing clinic, no pressure.”* (Interview 5)

Participants conveyed that *personal lifestyle factors* were influential in selecting a rural practice location. This included consideration of recreational activities, proximity to nature, the topography of the countryside, and a way of living in relation to the surrounding environment.

“*I love nature so there’s a lot of beluga whales, polar bears, the northern lights emerge, and there’s dog sledding, it’s more the recreational part of it that’s actually quite fun as well. So despite how cold it gets there, I found it just a very charming little place, and it kind of fits the lifestyle that I thought I would enjoy, something that was different than city life.”* (Interview 8)

*“…we’re cross-country skiers, love hiking, love backpacking, so the mountain area was good.”* (Interview 3)

## Discussion

Family physicians in rural practice from urban backgrounds made their rural career choice primarily during medical school or residency training. As such, our study emphasizes the role of educational systems on the career decision for rural practice. Substantive rural experiences during training appear to attract urban background family physicians to the broad scope and variety that rural practice offers, and to a personal lifestyle that is perceived to be simpler, less busy, and connected to nature and outdoor recreation. While there are some overlapping factors, this study differentiates between factors that can influence overall rural career choice and those that can influence the choice of a specific rural practice location.

A family physician’s decision for a particular rural practice location requires having a comfort level with specific group practice expectations. A new and important consideration is the right fit for not only rural lifestyle culture, but also rural professional culture of the community. While each rural community has its own culture, each medical practice evidently has its own clinical culture that a new physician joining the practice should align with. Familiarity with clinic operations, including work hours, call schedule, space availability, and management structure, as well as stepping into a location that has a practice-ready patient population, medical and professional support systems, and a rewarding income stream are all important considerations.

Our study identified that immersed exposure to rural community living, rural practice experience, and positive rural physician role models was influential for our urban background physicians deciding on rural practice. These factors support the continuation of appropriately structured rural rotations within urban-based family medicine residency programs. A study of rural physicians who graduated from Canadian medical schools found that rural exposure during medical school or residency training appears to be influential in the decision of urban background family physicians for rural practice and that interest in rural practice tends to increase as training progresses.^14^ Extended rural rotations or cumulative rural experiences over the span of the residency program likely serve to provide substantial exposure and comfort level to rural practice, rural lifestyle, and practice expectations, thus influencing rural career choice. However, it is not known if longer or more rural rotations increase rural intent or if rural intent leads to longer or more rural rotations;^25^ more likely there is a bi-directional effect. Residents should be supported in their interest in rural curricular experiences as this could offer an important opportunity to recruit urban background family physicians into rural practice.

Our findings suggest that factors that influence choice of rural practice could also inform the standards for evaluation of residency programs. Accreditation standards for family medicine residency programs specify that program evaluation should include “an evaluation of the quality of the different learning environment.”^26^ Significant exposure to a positive rural culture, including positive physician role models, operational management of the clinic, a spousal program, and available outdoor recreational activities, are aspects of program evaluation criteria that would be supported by our study findings.

Factors that influence rural practice have implications for rural physician recruitment strategies. Given that students from rural backgrounds currently represent a disproportionate minority of the enrollment in medical school,^27^ unless there are dramatic changes in admissions that impact medical school demographics, it is unlikely that rural background students alone will contribute significantly to resolve the rural physician shortage. Governments, rural communities, and rural-focused organizations seeking to recruit rural physicians must consider the possibility of recruiting learners of an urban background. An increased likelihood of recruitment may be realized simply by inviting the learner to return for additional rural medical education experiences. Longer or recurring rural rotations facilitate the development of supportive relationships with a mentor and likely have a positive influence on rural career choice. The recreational aspects of the community and surrounding area should also be emphasized to both the potential recruit and partner/spouse.

A strength of our study is the richness of the qualitative interview data and analyst triangulation. We used four analysts to review and code the data which facilitated multiple ways of interpreting the data and illuminating blind spots, as well as provided a check on selective perception. While in qualitative research we cannot prove transferability of research findings, we feel that the description of the study methods and the findings themselves establish that the findings may have applicability to other contexts and settings. Ultimately, it is incumbent upon the reader to make connections between the study elements and their own experiences in order to assess the degree of transferability.

The study is limited in its predominantly women and Canadian medical graduate participants. At the time of the study, 9-10 years had passed since some participants completed their residency program. While this may have introduced memory recall bias which would have likely tended in the direction of more experienced and thoughtful reflection on decision making and may not have truly captured all the factors considered at the time the decision for rural practice was actually made, it enabled participants to identify factors that influenced their career choice across the educational continuum, rather than at one point in the educational process. Also, participants identified factors that were perceived to have influenced rural practice decisions. Due to the descriptive nature of the study design, we are unable to ascertain the relative importance of these factors. It is possible that some factors identified by the study participants as being those that attracted them to rural practice, may be the same features that dissuaded other physicians from rural practice; therefore, the same factor may have the opposite effect on different individuals. For example, the broad scope and variety of rural practice may be challenging and professionally fulfilling to some, while others may consider it overwhelming and stressful. A rural lifestyle may be appealing to some, while others may find it isolating. As such, the push-pull effect of the factors on different groups is unknown. The definition of rural within the Alberta context may not be transferable to some settings, particularly those with concentrated populations and those not geographically dispersed. In order to refine rural recruitment strategies and provide input to rural educational initiatives, further understanding of urban background rural family physicians is needed. Similar studies should be conducted with physicians who have graduated from other programs. Examination of the acculturation of urban background physicians to rural culture is warranted. Defining the unique characteristics of positive rural physician role models would aid educational programs in selecting exceptional teaching sites, as well as provide guidance in terms of faculty development of rural preceptors. Inquiry into differences between rural and urban professional medical culture could further identify strategies for designing educational experiences aimed at attracting family medicine residents to rural practice.

## Conclusion

Practicing urban background family physicians in our study indicated that their rural career decisions were influenced by a mix of rural educational experiences, clinical practice considerations, and personal or lifestyle factors. There appears to be a role for the flexibility to provide extended or cumulative rural experiences in urban-based family medicine residency programs. Our study suggests that rural cultural competence could be considered an additional standard for the evaluation of residency programs. While there is some overlap in factors that influence rural career choice and choice of a particular practice location, familiarity and comfort level with operational aspects of the practice, are distinctive to choosing a practice location. Further understanding of practicing rural family physicians with an urban background is needed for physician resource planning.

## References

[1] Pong RW, Pitblado JR Geographic distribution of physicians in Canada: beyond how many and where. Ottawa: Canadian Institute for Health Information 2005.

[2] Ricketts TC The migration of physicians and the local supply of practitioners: a five-year comparison. Acad Med. 2013;88(12):1913-8. 10.1097/ACM.0000000000000012 24128618

[3] McGrail MR, Wingrove PM, Petterson SM, Bazemore AW Mobility of US rural primary care physicians during 2000-2014. Ann Fam Med. 2017;15(4):322-8 10.1370/afm.2096 28694267 PMC5505450

[4] Lee DM, Nichols T Physician recruitment and retention in rural and underserved areas. Int J Health Care Qual Assur. 2014:27(7):642-52. 10.1108/IJHCQA-04-2014-0042 25252569

[5] Brooks RG, Walsh M, Mardon RE, Lewis M, Clawson A The roles of nature and nurture in the recruitment and retention of primary care physicians in rural areas: a review of the literature. Acad Med. 2002;77(8):790-8. 10.1097/00001888-200208000-00008 12176692

[6] Henry JA, Edwards BJ, Crotty B Why do medical graduates choose rural careers? Rural Remote Health. 2009;9(1):1083 10.22605/RRH1083 19257797

[7] Parlier AB, Galvin SL, Thach S, Kruidenier D, Fagan EB The road to rural primary care: a narrative review of factors that help develop, recruit, and retain rural primary care physicians. Acad Med. 2018;93(1):130-40. 10.1097/ACM.0000000000001839 28767498

[8] Rural origins and choosing family medicine predict future rural practice. Am Fam Physician. 2007;76(2):207. 17695565

[9] Rabinowitz HK, Diamond JJ, Markham FW, Santana AJ The relationship between entering medical students' backgrounds and career plans and their rural practice outcomes three decades later. Acad Med. 2012;87(4):493-7. 10.1097/ACM.0b013e3182488c06 22361786

[10] Laven GA, Beilby JJ, Wilkinson D, McElroy HJ Factors associated with rural practice among Australian-trained general practitioners. MJA. 2003;179(2):75-9. 10.5694/j.1326-5377.2003.tb05439.x 12864716

[11] Myroniuk L, Adamiak P, Bajaj S, Myhre DL Recruitment and retention of physicians in rural Alberta: the spousal perspective. Rural Remote Health. 2016;16(1):3620. 26859245

[12] Woloschuk W, Crutcher R, Szafran O Preparedness for rural community leadership and its impact on practice location of family medicine graduates. Aust J Rur Health. 2005;13(1):3-7. 10.1111/j.1440-1854.2004.00637.x 15720307

[13] Woloschuk W, Tarrant M Do students from rural backgrounds engage in rural family practice more than their urban-raised peers? Med Educ. 2004;38(3):259-61. 10.1046/j.1365-2923.2004.01764.x 14996334

[14] Chan BT, Degani N, Crichton T, et al Factors influencing family physicians to enter rural practice: does rural or urban background make a difference? Can Fam Physician. 2005;51(Sep):1246-7. 16926939 PMC1479469

[15] Easterbrook M, Godwin M, Wilson R, et al Rural background and clinical rural rotations during medical training: effect on practice location. CMAJ. 1999;160(8):1159-63. 10234346 PMC1230268

[16] Potter JM Characteristics of Alaskan family physicians as determinants of practice location. Alaska Med. 1995;37(2):49-52,79. 7661326

[17] Stenger J, Cashman SB, Savageau JA The primary care physician workforce in Massachusetts: implications for the workforce in rural, small town America. J Rural Health. 2008;24(4):375-83. 10.1111/j.1748-0361.2008.00184.x 19007392

[18] Stagg P, Greenhill J, Worley PS A new model to understand the career choice and practice location decisions of medical graduates. Rural Remote Health. 2009;9(4):1245 Epub 2009 Nov 28. 10.22605/RRH1245 19943714

[19] Myhre DL, Bajaj S, Jackson W Determinants of an urban origin student choosing rural practice: a scoping review. Rural Remote Health. 2015;15(3):3483. 26391014

[20] Kane KY, Quinn KJ, Stevermer JJ, et al Summer in the country: changes in medical students’ perceptions following an innovative rural community experience. Acad Med. 2013;88(8):1157-63. 10.1097/ACM.0b013e318299fb5d 23807101

[21] Tolhurst HM, Adams J, Stewart SM An exploration of when urban background medical students become interested in rural practice. Rural Remote Health. 2006;6(1):452. Epub 2006 Mar 8. 16544958

[22] Myhre DL, Adamiak PJ, Pedersen JS Specialty resident perceptions of the impact of a distributed education model on practice location intentions. Med Teach. 2015;37(9):856-61. Epub 2014 Dec 19. 10.3109/0142159X.2014.993952 25523114

[23] Rourke JT, Incitti F, Rourke LL, Kennard M Relationship between practice location of Ontario family physicians and their rural background or amount of rural medical education experience. Can J Rural Med. 2005;10(4):231-40. 16356384

[24] Poole P, Stoner T, Verstappen A, Bagg W Medical students: where have they come from; where are they going? N Z Med J. 2016;129(1435):59-67. 27355169

[25] Hogenbirk JC, Mian O, Pong RW Postgraduate specialty training in northeastern Ontario and subsequent practice location. Rural Remote Health. 2011;11(1):1603. 21381861

[26] The College of Family Physicians of Canada. Specific standards for family medicine residency programs accredited by the College of Family Physicians of Canada. Mississauga, ON: The College of Family Physicians of Canada 7 2016.

[27] Society of Rural Physicians of Canada. Admission of rural origin students to medical school: recommended strategies. 5 2004 Available from: http://www.cfpc.ca/uploadedFiles/Resources/Resource_Items/Health_Professionals/AdmissionRuralOrigin.pdf [Accessed 2018 Jul 18].

